# Structures and Properties of the Self-Assembling Diphenylalanine Peptide Nanotubes Containing Water Molecules: Modeling and Data Analysis

**DOI:** 10.3390/nano10101999

**Published:** 2020-10-10

**Authors:** Vladimir Bystrov, Jose Coutinho, Pavel Zelenovskiy, Alla Nuraeva, Svitlana Kopyl, Olga Zhulyabina, Vsevolod Tverdislov

**Affiliations:** 1 Institute of Mathematical Problems of Biology, Keldysh Institute of Applied Mathematics, RAS, Pushchino, Moscow 142290, Russia; 2 Department of Physics & I3N, University of Aveiro, Campus Santiago, 3810-193 Aveiro, Portugal; jose.coutinho@ua.pt; 3 School of Natural Sciences and Mathematics, Ural Federal University, Ekaterinburg 620000, Russia; zelenovskiy@urfu.ru (P.Z.); alla.nuraeva@urfu.ru (A.N.); 4 Department of Chemistry & CICECO-Aveiro Institute of Materials, University of Aveiro, 3810-193 Aveiro, Portugal; 5 Department of Physics & CICECO-Aveiro Institute of Materials, University of Aveiro, 3810-193 Aveiro, Portugal; svitlanakopyl@ua.pt; 6 Faculty of Physics, Lomonosov Moscow State University, Moscow 119991, Russia; zhulyabina.o@yandex.ru (O.Z.); tverdislov@mail.ru (V.T.)

**Keywords:** diphenylalanine, peptide nanotubes, self-assembly, water molecules, DFT, molecular modelling, semi-empirical methods, polarization, chirality

## Abstract

The structures and properties of the diphenylalanine (FF) peptide nanotubes (PNTs), both L-chiral and D-chiral (L-FF and D-FF) and empty and filled with water/ice clusters, are presented and analyzed. DFT (VASP) and semi-empirical calculations (HyperChem) to study these structural and physical properties of PNTs (including ferroelectric) were used. The results obtained show that after optimization the dipole moment and polarization of both chiral type L-FF and D-FF PNT and embedded water/ice cluster are enhanced; the water/ice cluster acquire the helix-like structure similar as L-FF and D-FF PNT. Ferroelectric properties of tubular water/ice helix-like cluster, obtained after optimization inside L-FF and D-FF PNT, as well of the total L-FF and D-FF PNT with embedded water/ice cluster, are discussed.

## 1. Introduction

Self-assembly of complex molecular structures based on various amino acids (AAs) is one of the most important phenomena both in living nature and in nanomaterials development [1,2,3,4]. At the same time, it is now known that the chirality of the initial molecules plays an important role in self-assembly processes [5,6,7]. All this is important both for our understanding of the basic principles of the emergence of life, and for numerous practical applications [5,6,7], including the new nanomaterials synthesis for nanoelectronics [3,8,9] and biomaterials for usage in biomedicine [10,11], including targeted drug delivery [12,13].

One necessary research approach is computer molecular modeling of the processes of self-organization of molecular systems at different levels and by different methods [14,15,16]. All AAs have their own dipole moments [17], which interact with each other and self-organize into more complex molecular and crystalline structures, such as peptide nanotubes (PNTs) and similar nanostructures [18]. As results, many of these structures have piezoelectric and ferroelectric properties [19,20,21,22,23,24]. This has been shown and investigated in detail in our previous works [24,25,26,27,28,29,30]. Self-assembly of such PNTs occurs in aqueous media rather quickly and under certain conditions that affect the rate of their growth and the shape of self-organizing structures [31,32]. It is important that their structural and physical properties turn out to be dependent on the chirality of the original molecules of amino acids and dipeptides [29,30,31,32]. In many cases, water molecules also appear in the internal hydrophilic cavity of such PNTs [29,30,31,32,33,34,35,36]. They affect the physical properties of PNT and largely determine their changes. However, the experimental detection of water molecules by X-ray diffraction methods is very difficult. In this case, it is computer simulation methods that can help: they play an important role for a clearer identification of these water structures [37] and their study, establishing their structural and physical properties and their effect on the properties of PNT as a whole. In principal, computer molecular modeling allows us to calculate, investigate, and predict the basic physical properties of these nanostructures based on any various AAs, with and without including water molecules.

In this paper, we continue our further study of the structural and physical (including polar and ferroelectric-like) properties of PNTs based on diphenylalanine (FF) (FF PNTs) of different chirality—L-chiral (L-FF) and D-chiral (D-FF)—and focus on the embedded molecular clusters of water in the PNTs’ internal hydrophilic cavity. An optimum possible number of water molecules per one unit cell of the D-FF and L-FF hexagonal crystal structures was determined. These structural data and water molecules are also considered in molecular models for D-FF and L-FF PNTs, which have at least two coils of the PNT helix structure [25,28,29] that correspond to the period of the hexagonal unit cell along the c axis. The influences of these water molecules on PNT properties are analyzed, including a change in the dipole moments and polarization of the PNTs, as well as a change in the structure and properties (dipole moment and polarization) of water clusters embedded in a PNT cavity for both chirality types.

In all calculations, the quantum semi-empirical AM1, PM3, RM1 methods in the Hartee-Fock approximations (from the HyperChem package [38]) are used. The initial structural data of D-FF and L-FF from the crystallographic database [39] were taken, and for their calculations and optimization, the density functional theory (DFT) methods (in Vienna Ab initio Simulation Package (VASP) program [40]) are applied, taking into account the Van der Waals interactions (VdW correction by “PBE + D3” method was used, available in VASP). All results obtained in comparison with known and experimental data are analyzed.

## 2. Models and Computational Details

### 2.1. Main Methods and Software

The calculations were carried out using DFT methods, as implemented by VASP [40,41,42,43]. The exchange-correlation potential was evaluated using the generalized gradient approximation (GGA) according to Perdew, Burke, and Ernzerhof (PBE) functional [43,44]. Core states were described by means of the projector augmented wave method [45], while the Kohn-Sham task (for calculating the energies of the ground states of systems) was calculated using plane waves with kinetic energy up to Ecut = 400 eV to expand the wave functions.

In the calculations of self-assembled systems based on amino acids, it is also necessary to take into account the Van der Waals (VdW) interactions. A useful pragmatic method to work around this problem is to add a correction to the conventional Kohn-Sham DFT energy. Here, we used the “PBE + D3” method for VdW correction developed by Grimme S., Antony J., Ehrlich S., and Krieg S. [46] and named D3. This method is compatible with PBE and is implemented in VASP (PBE + D3).

**Figure 1:**
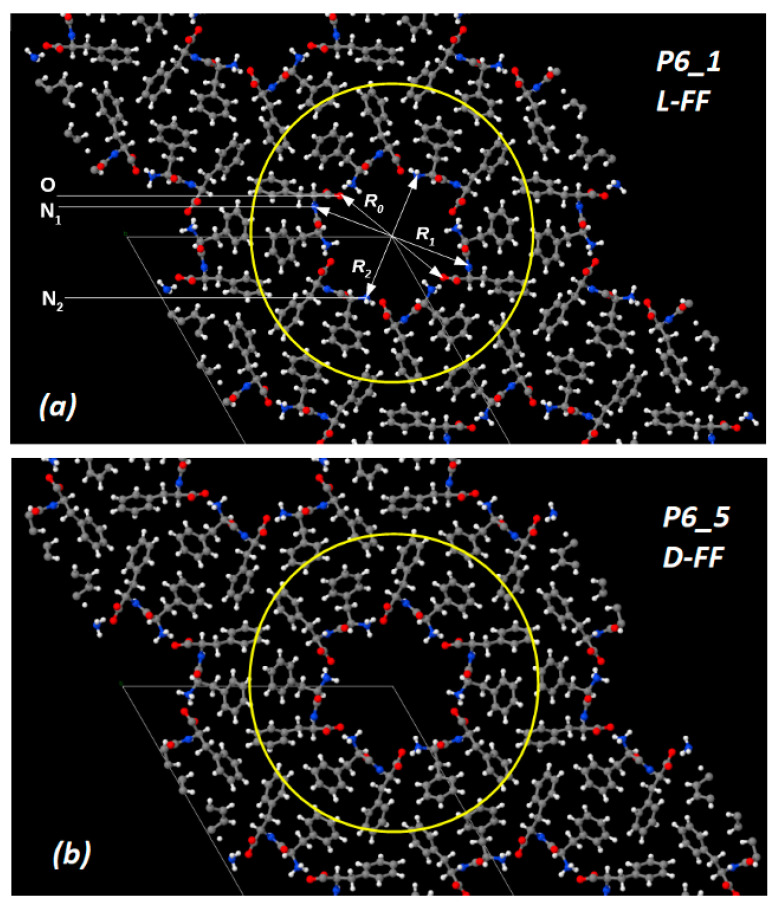
Images of structures based on the Cambridge Crystallographic Data Center (CCDC) for two different diphenylalanine (FF) enantiomers with symmetry elements (obtained using Jmol after performing Vienna Ab initio Simulation Package (VASP) calculations) for cases: (**a**) L-chiral (L-FF) with space group P6_1_ and (**b**) D-chiral (D-FF) with space groups P6_5_. Hexagonal cells are marked by thin lines. Selected atoms and molecules for the formation of peptide nanotubes (PNTs) are shown by a circle in yellow lines. Atoms are marked with colors: oxygen—red, nitrogen—blue, carbon—gray, hydrogen—white. The upper figure (**a**) shows the distances in the inner cavity: R_0_—between the oxygen atoms O…O; R_1_—between the nitrogen atoms N_1_...N_1_ (larger “far” diameter PNT); R_2_—between the nitrogen atoms N_2_...N_2_ of the opposing NH_3_^+^ groups (short “near” inner diameter PNT).

In this paper, further study of diphenylalanine peptide nanotubes is based on the models constructed from experimental data of their crystallographic structures obtained by X-ray methods and recorded in the crystallographic database of the Cambridge Crystallographic Data Center (CCDC) [39]. (These data correspond to No. CCDC 16337 for L-FF [33] and No. CCDC 1853771 for D-FF [25,29]). These structural data and visual models of both PNT L-FF and D-FF structures are easily reproduced in accordance with their periodic crystallographic cell parameters (see Table 1) in various software systems (such as “Mercury”—https://www.ccdc.cam.ac.uk/solutions/csd-system/components/mercury/— a system for visualization and analysis of crystallographic structures compatible with the CCDC base). Using all these data, the source files were built up for modeling and calculations based on DFT methods in the VASP program [40] (see Figure 1).

Then, these structures were also converted and transferred into files in the HyperChem [38] workspace for their further analysis and calculations of their polar properties with various quantum-mechanical semi-empirical methods.

### 2.2. Models of Initial Water-Free Crystal Structures

The initial and water-free crystal structures of both L-FF and D-FF PNT types are shown in Figure 1. The anhydrous and non-centrosymmetric hexagonal unit cell for both enantiomers crystal structures contains six FF molecules, and it is formed by a total of 258 atoms, while the space groups of these enantiomers are different: P6_1_ space group for L-FF and P6_5_ for D-FF. These initial crystal configurations were obtained from X-ray experimental Cambridge Crystallographic Data Center (CCDC) data [39]. In all cases, the Brillouin zone (BZ) was sampled using a Monkhorst-Pack [47] scheme with a 1 × 1 × 3 mesh k-point sampling. The Hartree-Fock exact exchange was evaluated using the same k-point grid computed for the DFT potential. This enables a real-space grid of 120 × 120 × 28 points to keep along a1, a2, and a3 lattice vectors, respectively, which corresponds to the experimental lattice constants a1 = a2 = *a* = *b* ~24 Å and a3 = *c* = ~5.44 Å of the D-FF crystal structures data (Table 1). The relevant grid density appears to be about 5 points/Å along all the three directions.

The relaxation (optimization of the total energy) of both initial structures was carried out, and the same procedure was also performed for all cases of simulated structures with different numbers of water molecules in the cavity of the inner channel of the nanotubes. The relaxation cycle was stopped when the maximum force acting on lattice vectors and ions became less than 10 mV/Å. The main method used here for finding the minimum total energy in VASP [40] is the conjugate gradient algorithm; but in some cases, we used a different algorithm for the case close to a local minimum. For better optimization, in some cases, we varied the maximum acting force limit.

### 2.3. Model of Water/Ice Clusters

The influence of water molecules on D-FF and L-FF PNT properties was studied with the use of the hexagonal ***Ih*** ice cluster model [48], which served as the basis for the construction of the initial model of a water cluster with different numbers *n* of water molecules (in the case of small n*(H_2_O) water clusters with *n* = 2, 4, 6, etc., only a short part of such ***Ih*** ice cluster was used)). The model clusters constructed were introduced into the cavity of the initial anhydrous nanotubes, as was done in our previous recent work [26]. Then, the whole D-FF and L-FF PNTs structures, filled with this embedded water/ice cluster with *n* water molecules, was optimized, keeping the lattice parameters *a, b, c*, of the initial nanotubes constant (Table 1). This is necessary to obtain a correlation with the initial experimental data. The obtained optimized structures with different numbers of water molecules in their cavity were collected and stored for further analysis of their parameters and visual control using different methods.

### 2.4. Estimation of Interaction Energy of Water/Ice Cluster and PNT

To estimate the energy of water molecule interaction *E_i_* with PNT for each optimized structure with different quantities of water molecules *n*, we calculated a change in the total energy as the number of water molecules (average energy per FF unit cell) as a function of the number of water molecules increased:

(1) $$E_{i}=E_{tot}–E_{PNT}-n\cdot E\left(H_{2}O\right)$$

where *E_tot_* is the total energy per unit cell for the optimized PNT structure with water molecules, *E_PNT_* is the energy of the optimized PNT structure without water molecules, *E*(H_2_O) is the energy of a relaxed single water molecule H_2_O, and *n* is the number of H_2_O molecules used in the calculation. This approach is similar in general to that of Ref. [36] (but differs in some computational details). The *E_i_* obtained here is the average interaction (or binding) energy that provides information concerning the interaction between water molecules and their surroundings, i.e., the sum interaction between water molecules and the inner wall of the nanotube via hydrogen bonds (HBs) and the intensity of the intermolecular HB interactions formed between water molecules [49,50,51]. Naturally, with an increase in the number of water molecules in the inner cavity of the nanotube, this energy also changes.

To determine changes in the main distances (R_1_, R_2_) inside the PNT cavity after relaxation and optimization of all the structures, we use the *Jmol* software tool for visual presentation of all the structures extracted after calculations. For extraction of all atomic files and transformation of their formats, the *OpenBabel* and *Cyberduck* software tools were used too.

### 2.5. Semi-Empirical Calculations

To obtain the values of the energy, dipole moment, and polarization of the optimized D-FF and L-FF structures with and without water and individual extracted water clusters, the HyperChem package [38] was used with various quantum-mechanical semi-empirical AM1 (Austin Model 1, developed by Dewar M.J.S., et al. [52,53]), PM3 (Parametrization Method No. 3, is a reparameterization of AM1, developed by Stewart J.J.P., et al. [53,54,55,56,57]), RM1 (Recife Model 1, is a reparameterization of AM1, developed by Rocha G.B., et al. [58,59]) methods (the restricted Hartree-Fock (RHF) approximation was used). For this purpose, the optimization of 21 water molecules embedded in D-FF and L-FF cavity with fixed unit cell parameters (corresponding to the experimental data [25,29,33,39]) was performed. Only these water molecules were optimized, whereas atomic positions for all other atoms of FF molecules were kept “frozen”. The optimized water cluster structures were extracted from the D-FF and L-FF cavity to be investigated independently using *OpenBabel*, *Cuberduck*, and HyperChem software [38]. Scheme of the main steps of these procedures are presented in Figure 4 (see below and more details in the next section).

Thus, the optimized structures were transformed from periodical crystal structures in VASP to HyperChem workspace as one, two, and more coil helix molecular structures, where all further calculations were carried out.

## 3. Results and Discussions

### 3.1. Determination of the Optimal Number of Water Molecules in the PNT Cavity

To investigate the presence of water molecules and find their optimal number, the PNT structures (both D-FF and L-FF) with different numbers of water molecules were calculated and optimized. As result, optimized PNT models containing different number of water molecules in the inner cavity of the PNTs were obtained, and the dependence of PNT properties on the number of water molecules was studied. Note that optimizing L-FF PNTs with water is concerned with difficulties since these structures are less stable as compared to D-FF PNTs. This also agrees with previously obtained data: D-FF nanotubes have a denser and stronger deep packing than L-FF PNTs [25,28,29]; L-FF PNTs have larger cavity sizes, with looser and less uniform surface of the internal cavity compared to D-FF PNTs [28,30]. It was necessary to vary some parameters and methods of the optimization procedure in VASP to obtain the most stable and suitable optimized structure. These calculations required more time to proceed on a computer cluster. Nevertheless, it was possible to obtain good optimized structures for both D-FF and L-FF and choose the better ones for further analysis.

**Figure 2:**
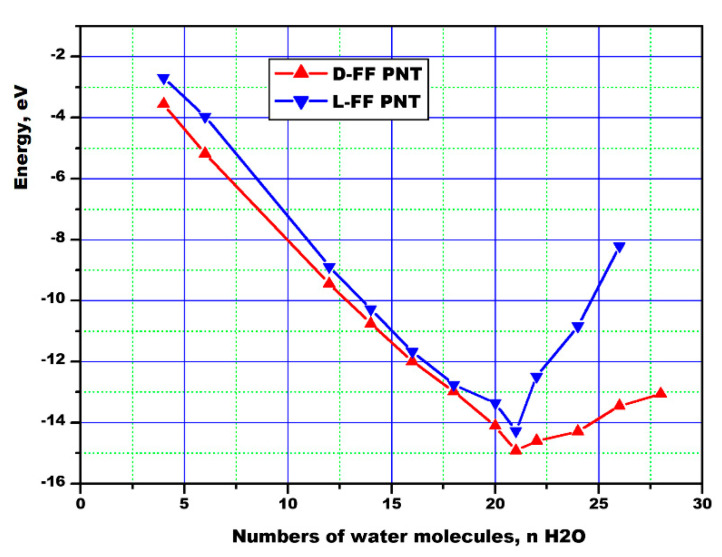
Average interaction energy *E_i_* between water and PNT as a function of number of water molecules confined in the hydrophilic channel of FF PNTs for each chirality types: L-FF and D-FF.

As a result, the dependence of the average interaction energy *E_i_* (1) per one unit cell FF as a function of the number of water molecules *n* were obtained for both types of structures L-FF and D-FF PNTs, convincingly showing that the minimum of this energy *E_i_* is observed for *n* = 21 (Figure 2). The peculiarity of the behavior of these dependence (for both chirality type L-FF and D-FF), the values and position of the minimum of energies turned out to be similar to the data [36] and are comparable in magnitudes: the value of the energy is in order of ~−14 eV in the minimum position, corresponding for 21 H_2_O, which is the same as in our case.

The calculated water-PNT interaction energy *E_i_* is the energy of an interaction between the water molecules and the inner wall of the nanotube through the hydrogen bonds (HBs) [49,50]. The formation of a network of HBs between water molecules and the nearest hydrophilic oxygen and nitrogen atoms at the inner surface of the nanotube cavity was found for all optimized D-FF and L-FF structures (for more details, see discussion below in Section 3.2).

These results are also confirmed by the calculated dependence of the internal diameters of the nanotube cavity, determined by the distances between the main nitrogen atoms N_1_, N_2_: R_1_ (N_1_-N_1_) and R_2_ (N_2_-N_2_) (these distances were determined in Figure 1a). As the number of water molecules *n* increases, the size of the inner PNT cavity changes for both types of chirality L-FF and D-FF. Comparing calculated and experimental values of R_1_ and R_2_, we can see that, when the number of molecules is equal to *n* = 21, the calculated curves intersect with the experimental values, which are according to X-ray structural data for the initial nanotubes (see data in Table 2), for both chirality type L-FF and D-FF PNTs.

**Figure 3:**
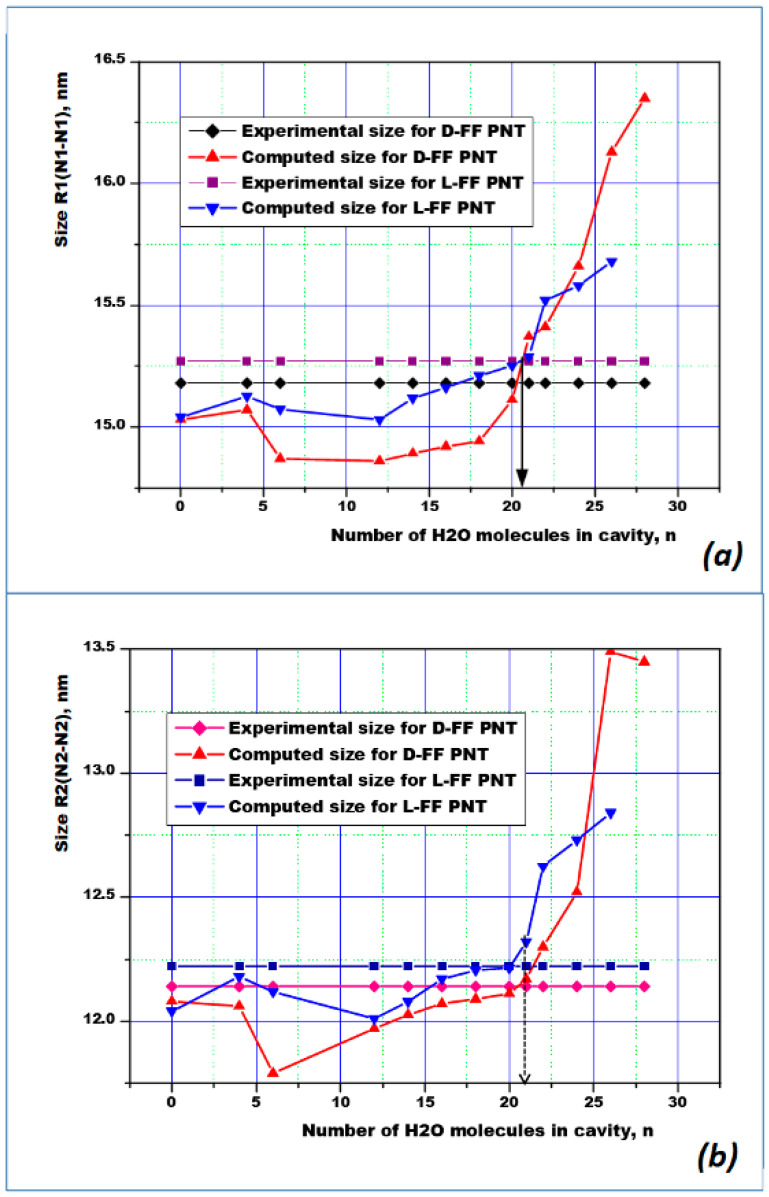
Dependences of the internal cavity sizes of the optimized structures of nanotubes of both types of chirality L-FF and D-FF containing a different number of water molecules on the number n of these water molecules, in comparison with the known sizes of the internal sizes of nanotubes according to experimental data (Table 2) [25,29,33,39]: (**a**) R_1_—between the nitrogen atoms N_1_...N_1_ (larger “distant” diameter PNT); (**b**) R_2_—between the nitrogen atoms N_2_...N_2_ of the opposite NH3^+^ groups (short “near” inner diameter PNT).

These graphs (Figure 3a,b) show that *n* = 21 and the obtained curves of the dependencies of the internal dimensions of the cavities of the optimized structures of the nanotubes of both chiral types coincide in very close sizes variations with the experimental data.

As a result, it can be argued that in the L-FF and D-FF cavities of both types of chirality there are about *n* = 21 water molecules per unit cell of these FF periodic molecular crystal structures.

Preliminary analysis showed that, in all cases, water-ice clusters after optimization in the internal cavity of both types L-FF and D-FF PNT change their structure and properties. However, the case with the found amount of 21 water molecules is of greater interest, which corresponds to the minimum total energy of both types of chirality. Extraction of the optimized water cluster (with 21 H_2_O molecules) from the inner cavity shows that this cluster has changed as compared to the initial hexagonal ice structure and acquired a helix-like structure, close to the helix, which is typical for L-FF and D-FF PNTs per se (see details below in Section 3.3). Moreover, a water cluster splits into inner and outer parts, and the latter actively forms hydrogen bonds with the atoms of nitrogen and oxygen of the inner surface of the PNT cavity. All these data need more deep and detailed analysis.

### 3.2. Water Cluster Structures Details

**Figure 4:**
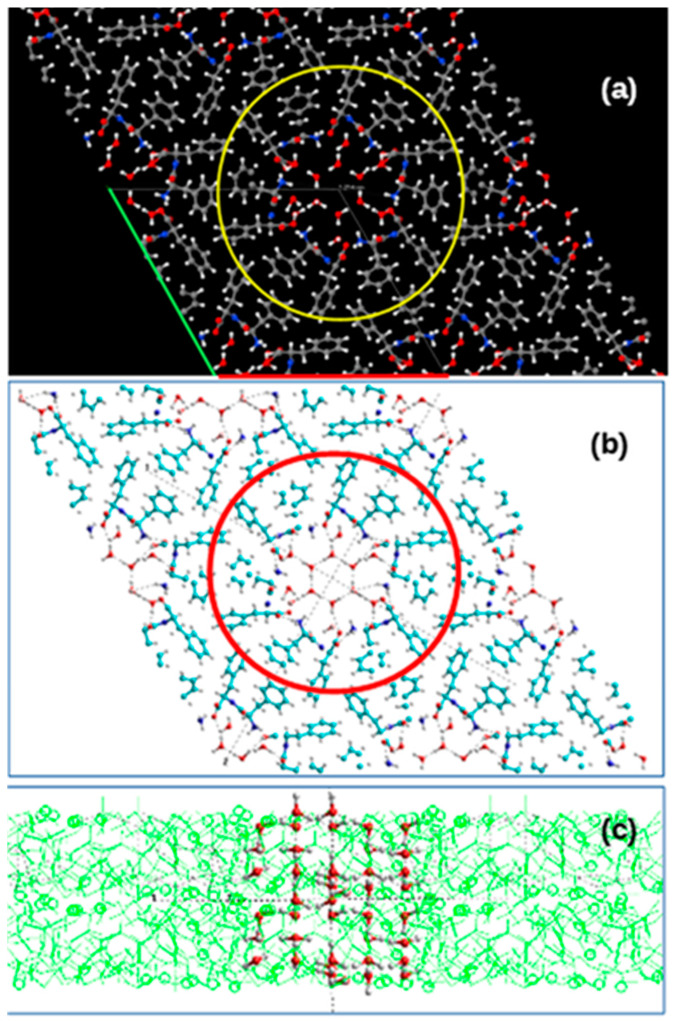
Schematic images of the initial water/ice cluster embedded into the inner hydrophilic cavity of the D-FF PNT: (**a**) top Z-projection images consisting from four unit cells of D-FF crystal structure obtained using *Jmol* from VASP initial data before optimization calculations (green and red lines marked the unit cell; yellow circle shows the selected atoms and molecules that form the PNT with cavity in the center filled with water/ice cluster molecules; the colors of atoms are the same as in Figure 1); (**b**) the same Z-projection images converted from VASP to HyperChem workspace using *Cyberduck* and *OpenBabel* software (red circle shows the same selected atoms and molecules that form the PNT with cavity in the center filled with water cluster molecules; the colours of atoms here are—Carbon is Cyan, Red is Oxygen, White (or Gray) is Hydrogen); (**c**) side Y-projection and cross-section of D-FF structures with selected water molecules formed initial water cluster.

Let us consider the structures of water clusters obtained with 21 H_2_O molecules per a unit cell in greater detail. In this case, the initial water cluster based on the hexagonal ***Ih*** ice cluster models and consisting from 21 H_2_O molecules per unit cell is presented on Figure 4 for the case of D-FF PNT. A similar initial structure is also used and for L-FF PNT. During the optimization process (using the VASP program, as described above), the structure of water clusters changes—there is a displacement of water molecules inside the cavity under the influence of an electric field inside the cavity (arisen from fixed FF dipoles, which create total strong polarization here and, accordingly, a strong electric field appears along the axis of the nanotube [24,25,26,27,28,29]).

**Figure 5:**
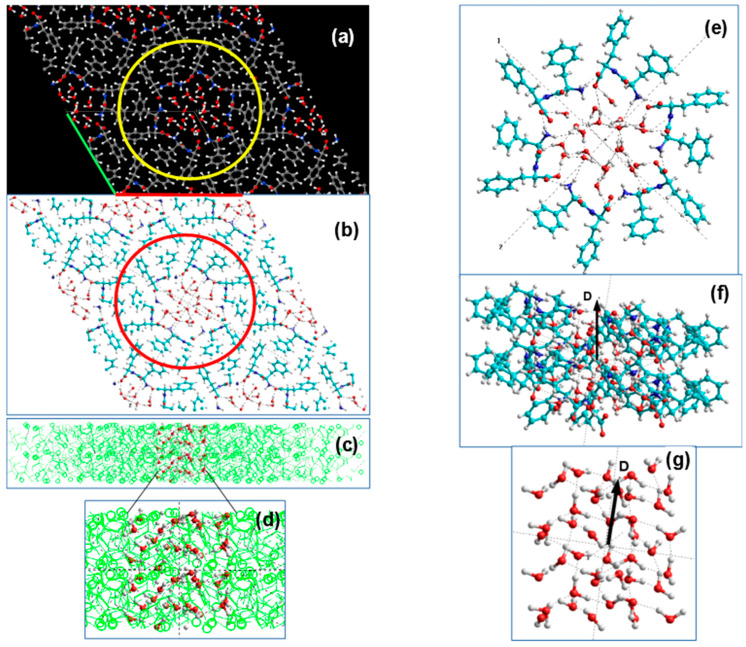
Schematics of the D-FF PNT structures with optimized 21 H_2_O per unit cell embedded water cluster (the designations used are the same as in Figure 4): (**a**) Z-projection image from VASP computed data with four unit cells; (**b**) the same image converted into HyperChem workspace; (**c**) side Y-projection and cross-section of D-FF structures with selected water molecules formed initial water cluster; (**d**) inset with biggest image of water cluster; (**e**) Z-projection image of the D-FF 2 coils with 42 H_2_O embedded water cluster; (**f**) Y-projection image of the D-FF 2 coils with 42 H_2_O embedded water cluster (**D** show the total dipole momentum); (**g**) Y-projection image of the 42 H_2_O water cluster extracted from D-FF PNT after optimization (**D** shows the total dipole momentum for water cluster).

**Figure 6:**
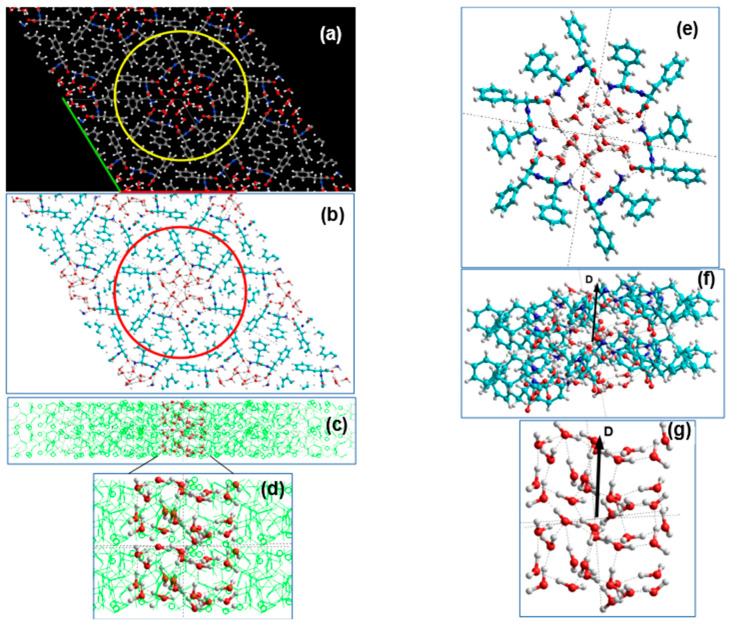
Schematics of the L-FF PNT structures with optimized 21 H_2_O per unit cell embedded water cluster (the designations used are the same as in Figure 4): (**a**) Z-projection image from VASP computed data with four unit cells; (**b**) the same image converted into HyperChem workspace; (**c**) side Y-projection and cross-section of L-FF structures with selected water molecules formed initial water cluster; (**d**) inset with biggest image of water cluster; (**e**) Z-projection image of the L-FF 2 coils with 42 H_2_O embedded water cluster; (**f**) Y-projection image of the L-FF 2 coils with 42 H_2_O embedded water cluster (**D** show the total dipole momentum); (**g**) Y-projection image of the 42 H_2_O water cluster extracted from L-FF PNT after optimization (**D** shows the total dipole momentum for water cluster).

As noted above, we optimize only the structure of the water cluster inside a PNT cavity, keeping fixed (“frozen”) atomic positions of all the atoms of all the FF molecules and the unit cell parameters of the PNT crystal structures. In this case, a rearrangement of hydrogen bonds occurs both between water molecules and between water molecules and FF molecules (in particular, with nitrogen and oxygen atoms of FF molecules on the inner hydrophilic surface of the nanotube’s cavity). This happens both in D-FF PNTs and in L-FF PNTs, but in different ways in accordance with their different internal structure and chirality. As a result, we get two altered and significantly different structures of water clusters after their optimization in D-FF and L-FF chirality cases (Figure 5 and Figure 6).

The initial water cluster with 21 H_2_O molecules constructed on the basis of an ***Ih*** hexagonal ice structure in general had a correct symmetrical organization and a small dipole moment directed on average perpendicular to the axis of the nanotube (see Table 3 and Table 4). After optimization of this cluster embedded in the cavity of both PNT, its structure has changed. Moreover, this happened in different ways, depending on the type of the surrounding PNT structure. In both cases (L and D), a distortion of the structure occurs with the formation of a strong dipole moment oriented along the PNT axis.

For a more detailed analysis, we transformed the optimized structures obtained from VASP into HyperChem workspace and distinguished between the models of the structures of both D-FF and L-FF PNTs themselves (consisting of two helix coils) and models of nanotubes containing 42 H_2_O molecules each. These models correspond to two layers extracted from the VASP structures with a super-cell of their four initial crystal unit cells in plane (and 8 = 2 × 2 × 2 unit cells total) for each D-FF and L-FF structures. Figure 5 and Figure 6 schematically show the procedures for such transformations. Then, we identified the structures of extracted water clusters themselves from the structures of nanotubes in the HyperChem workspace. This is also shown in Figure 5 and Figure 6.

**Figure 7:**
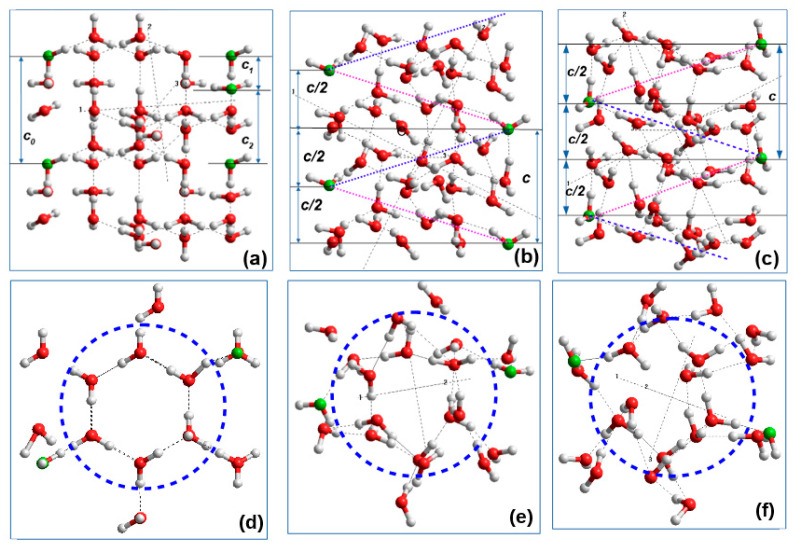
Extracted 42 H_2_O water clusters in Y-projection (**a,b,c**) and Z-projection (**d,e,f**): (**a**) and (**d**) for initial water cluster based on ***Ih*** structure; (**b**) and (**e**) after optimization within the D-FF PNT; (**c**) and (**f**) after optimization within the L-FF PNT. Dashed lines (with two colors: red and blue) on (**b**) and (**c**) show different direction of the helix-like structure water/ice cluster formation inside the D-FF PNT and L-FF PNT cavity cases. Dashed circles on (**d**), (**e**), and (**f**) show difference between inner and outer water/ice molecules groups. Parameters of periodical water/ice clusters structures are (**a**) *c*_0_ = 5.434 Å, *c*_1_ = 1.670 Å, *c*_2_ = 3.764 Å; (**b**) *c* = 5.441 Å; (**c**) *c* = 5.456 Å—these both correspond to the unit cell parameter *c* in D-FF and L-FF.

Figure 7 shows 42 H_2_O water clusters extracted from two-coil PNTs (initial and optimized in the D-FF and L-FF PNT). The main feature is that the shift of the individual water molecules is different for D-FF and L-FF PNT—they acquired a helix-like structure with a helix pitch equal to the period of the corresponding D-FF and L-FF PNT helix (that is equal to the period of their periodical crystal structure parameter *c*—see Table 1). Figure 7b,c schematically shows various helix-like structures (in Y-projection) in comparison with the initial water cluster structure (Figure 7a). Figure 7d–f shows a separation of the molecular groups of the water cluster into their internal (or inner) and external (or outer) sub-groups. The outer molecules interact actively with FF atoms on the inner hydrophilic PNT cavity surface, namely with nitrogen and oxygen atoms due to hydrogen bonds.

A more detailed analysis of the optimized water structure (particularly, for the D-FF PNT structure with 21 water molecules in the cavity) showed that water molecules near the cavity wall arrange approximately halfway between the layers of PNT. These water molecules have the strongest HBs with COO^−^ and NH3^+^ groups of PNT. This result was confirmed recently by a dielectric spectroscopy study [51]. These results are also confirmed by visual-differential analysis [30], which are to be published in a separate article.

### 3.3. Polarization Details

Let us now consider in greater detail the polar properties of both nanotubes with integrated water clusters and the water clusters themselves. Energetic and polar properties (dipole moments and polarization) of empty and filled with 21 water molecules per a unit cell D-FF and L-FF PNTs as well as the extracted water clusters separately were studied using quantum-chemical calculations based on semi-empirical quantum methods AM1, RM1, and PM3 in the restricted Hartree–Fock (RHF) approximation. The data obtained (energies, dipole momentum, polarization) as well the volumes of all molecular structures were presented in Table 3 and Table 4.

Accurate calculations are possible with HyperChem using methods that neglect some, but not all, of the electron-electron interactions. These methods are called neglect of differential overlap (NDO) methods. These methods were further improved and developed by Stewart et al. [52,53,54,55,56,57,58,59], and they are based on the so called “neglect of diatomic differential overlap (NDDO)” approximation, with several modifications and with the choice of a wide number of parameters enables one to reproduce experimental quantities. NDDO retains all one-center differential overlap terms when Coulomb and exchange integrals are computed. The NDDO approximation is the basis for the Modified Neglect of Diatomic Overlap (MNDO), Austin Model 1 (AM1), and Parametrization No. 3 (PM3) methods. AM1 is a modified MNDO method proposed and developed by Dewar et al. at the University of Texas at Austin [52,53,54]. AM1 is generally the most accurate computational method and is often the best method for collecting quantitative information [52,53,54]. PM3 differs from AM1 only in the values of the parameters [55,56,57]. The parameters for PM3 were obtained by comparing a large number of experiments with calculation results. As a rule, non-covalent interactions in the PM3 method are less repulsive than in AM1. Recife Model 1 (RM1) is another semiempirical method that was parameterized to calculate dipole moments and enthalpies of formation, with errors smaller than those for AM1 and PM3 [58,59]. Energetic properties of organic compounds can be calculated in both forms: isolated, and in solvent medium. All methods are available and provide close data for the biomolecular systems studied in this article based on C, O, N, and H atoms.

The total energy per a unit cell, *E_t_* (or energy of two-coils PNT models, shown in Figure 5 for D-FF and on Figure 6 for L-FF models), and the binding energy, *E_b_* (calculated here as a difference of total energies of the structure at the optimal distances between all elements *E_t_* (r ~ r_opt_) and energy of all its elements removed away at the infinite distance one from another *E_t_* (r ~ ∞), were calculated before and after water optimization (Table 3 for D-FF and Table 4 for L-FF PNT models).

These energies were calculated automatically by HyperChem software when we calculate any molecular structure by quantum semi-empirical methods in the single point (SP) option or in the geometry optimization option.

For the extracted water clusters before and after the optimization, the variations of the total Δ*E_t_* and binding Δ*E_b_* energies are the same (Table 3 for D-FF it is Δ*E_t_* ~ Δ*E_b_ ~* −87.15 eV and Table 4 for L-FF it is Δ*E_t_* ~ Δ*E_b_ ~* 84.43 eV, see in columns 2 and 3), thus demonstrating that changes in energy are only due to the realignment of water molecules in the cluster and changes in VdW interaction between water molecules and reorganization of the HB network structure. At the same time, for both D-FF and L-FF PNT with water cluster inside, the *E_t_* and *E_b_* energy decreased: (1) it is at around ~ −167.75 eV after the optimization for D-FF that is twice higher than that for the extracted water cluster ~ −87.15 eV (see Table 3, columns 5 and 6); (2) and for L-FF from ~ −164.66 eV up to ~ −84.43 eV (see Table 4, columns 5 and 6). This can mean that the formation of HBs between the water cluster and inner surface of a nanotube takes twice as much energy as merely a rearrangement of bonds inside the water cluster. In principle, this corresponds to the approaches in references [49,50], but we obtain here some new important quantitative data.

**Figure 8:**
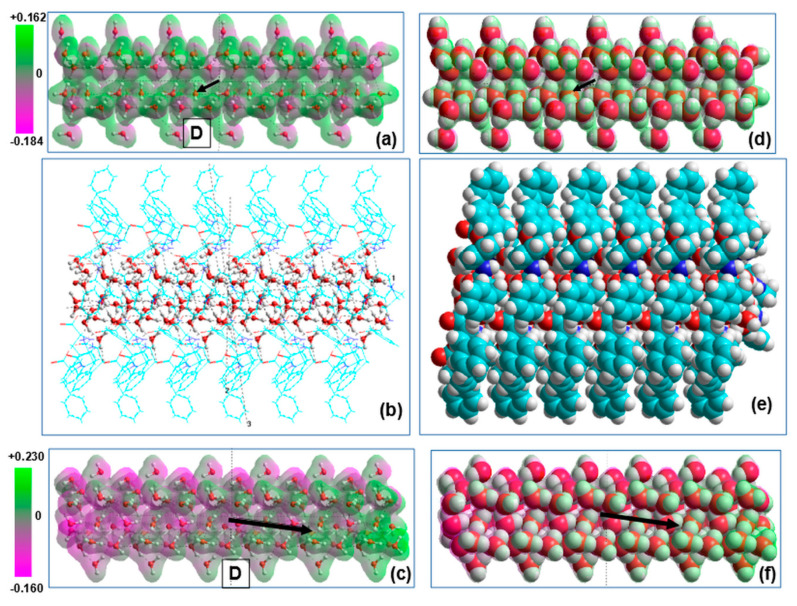
Model images of the water/ice tubular nanostructures and D-FF PNT with filled hydrophilic cavity, consisting from six repeated coils along the c-axis (in X-projection): (**a**) initial tubular water/ice structure, (**b**) D-FF PNT with embedded water/ice structures under optimization process, (**c**) water/ice tubular helix structure after optimization inside D-FF PNT, (**d**), (**e**), and (**f**) the same in the VdW surface presentation. Vector **D** show the direction of the total dipole momentum in the initial (D ~ 1 Debye) and optimized water/ice cluster (D ~ 97 Debye). Translucent 3D-mapped isosurface on (**a**) and (**c**) (as well as on (**d**) and (**f**)) illustrates the re-distribution of the electrostatic potential of water/ice cluster before and after optimization within D-FF PNT.

**Figure 9:**
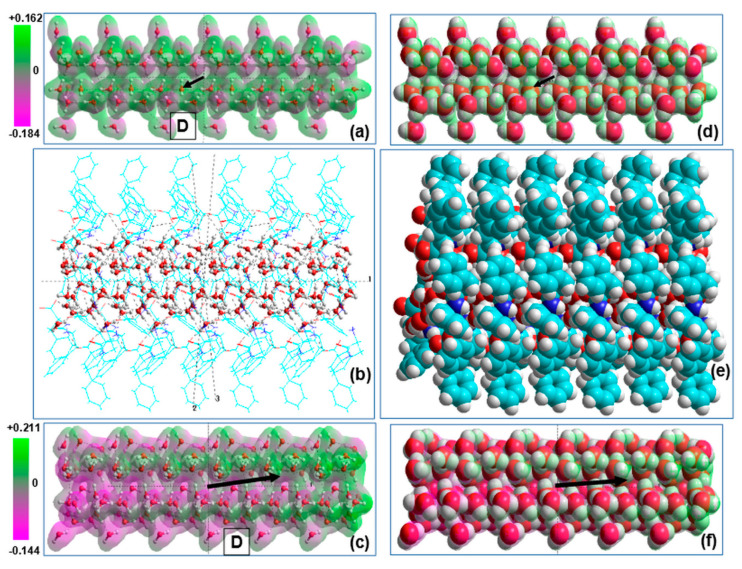
Model images of the water/ice tubular nanostructures and L-FF PNT with filled hydrophilic cavity, consisting from six repeated coils along c-axis (in X-projection): (**a**) initial tubular water/ice structure; (**b**) L-FF PNT with embedded water/ice structures under optimization process; (**c**) water/ice tubular helix structure after optimization inside l-FF PNT; (**d**–**f**) the same in the VdW surface presentation Vector **D** show the direction of the total dipole momentum in the initial (D ~1 Debye) and optimized water/ice cluster (D ~97 Debye). Translucent 3D-mapped isosurface on (**a**) and (**c**) (as well as on (**d**) and (**f**)) illustrates the re-distribution of the electrostatic potential of water/ice cluster before and after optimization within L-FF PNT.

The main results obtained were (1) after optimization the embedded water molecular cluster has a big own dipole moment strongly oriented along D-FF and L-FF PNT channel, while before it did not have such a defined orientation and a very small dipole moment (Figure 5, Figure 6f,g, Figure 8 and Figure 9 as well as Table 3 and Table 4); (2) the structure organization of both these water clusters after optimization inside D-FF and L-FF cavity has changed greatly—both have acquired helix properties, with the same helix step as D-FF and L-FF PNT (Figure 7); (3) the total dipole moment and polarization of both D-FF and L-FF PNT after optimization with embedded 21 H_2_O water/ice clusters enhance in the direction of main c-axis of each PNTs (Table 3, Table 4 and Table 5 and Figure 8 and Figure 9). Important feature is that after optimization inside D-FF and L-FF PNTs cavity the water/ice clusters acquired different directions of rotation of a helix. This property of helix lines as known is called chirality [6,7]: “right chirality” (D—from the Latin “dextra”) and “left chirality” (L from the Latin “laeva”).

This last result (showing in Figure 7, Figure 8 and Figure 9), concerned with the very pronounced realignment of water cluster structure, is confirmed by some other important computed data. The energy changes show that the main energy shift is concerned with changes of the VdW and HB energies of water molecule structures. It is clearly seen from changes in the extracted water molecules clusters obtained after optimization and their energies Δ*E_t_* and Δ*E_b_*, as was discussed above.

It is interesting to note that water molecules embedded into a carbon nanotube under the influence of high pressure and temperature lead to a formation of a similar helix structure [60]. It is known that the water confined to nanopores is investigated not only in carbon nanotubes, but in other nanoporous structures, for example, in boron nitride nanotubes (BNNTs) using first-principles calculations [61]. Another study of the polar property, polarization, and even the ferroelectricity in the ice-type (or water-type) nanostructures has recently been performed in nanoporous silicate materials, which have an ordered system of narrow cylindrical pores [62]. It is assumed that, in such filamentous pores of the studied materials, ferroelectric ice XI is formed.

We do not yet insist directly on the occurrence of ferroelectricity of water and ice structures inside peptide nanotubes, since reliable phase transitions between the polar (ferroelectric) and nonpolar (paraelectric) phases, which have pronounced changes in the dielectric constant according to the well-known Curie-Weiss law [19], have not yet been detected. Meanwhile, ferroelectricity was observed by Bdikin I., Bystrov V., Kopyl S., et al. in [63], though in other β-sheet FF PNT structure [17,24], not in α-helix, as is studied now [25,26,27,28,29]. Besides, water was not known to occur in the internal cavity of such PNT. This is to be clarified in the future, and possible results will be achieved with more detailed dielectric measurements [51]. Otherwise, the presence of such a significant and strictly oriented polarization clearly indicates the possibility of the existence of the ferroelectric-like phenomena here.

Returning to the first important result, it should be noted, that the extracted water clusters after optimization have the helix nanotube structure similar D-FF and L-FF PNT and possesses high of dipole moments and corresponding polarization value directed along nanotube OZ axis. The total dipole moment *D_t_* =|**D|** increases from ~1 D for the initial structure up to an order of ~29 D after the optimization for both chirality D-FF and L-FF (see Table 3 and Table 4, columns 2 and 3). The *D_t_* *=|***D|** of the water-filled D-FF nanotube also increases after the optimization from ~139.5 D up to ~158.5 D (Table 3, columns 5 and 6), and, similarly, from ~133.1 D up to ~157.8 D for L-FF PNT (Table 4, columns 5 and 6). The dipole moment found for empty PNT is about ~140.3 D for D-FF and ~140.8 D for L-FF, which are close to the values obtained in previous works for similar empty PNT [22,23,24,25,26,27,28,29]. For the filled PNT, the absolute value of the projection of the dipole moment at the nanotube axis, *D_z_*, is almost the same as *D_t_*, thus demonstrating that the dipole moment is oriented strongly along the D-FF and L-FF PNT axis. At the same time, the dipole moment of the extracted water cluster is shifted at about 15° from down the axis for D-FF PNT (Figure 8c), while for L-FF it is shifted at about 10–15° up from the L-FF PNT axis (Figure 9c). A water nanotube acquires such polar properties under the influence of D-FF and L-FF PNT—it is self-consistent and self-organized process modulated and induced by an electric field from the strongly oriented dipole moments of D-FF and L-FF. However, this effect is different for each chirality type.

The total polarization, *P_t_*, of D-FF and L-FF PNT with water molecules in a cavity (as well polarization of water clusters) can be calculated as a dipole moment per volume of the unit cell (in SI units *P_t_* [C/m^2^] = 3.33556255·*D_t_* [D]/*V* [Å^3^]). The values of the VdW volume of studied water clusters, D-FF and L-FF PNT cells, as well corresponding *P_t_* values are presented in Table 3, Table 4 and Table 5. Since water inside the nanochannel increases the volume of the unit cell (as was demonstrated by DFT calculations), the value of *P_t_* for filled D-FF PNT after the optimization may be somewhat less than that of the empty PNT. So, for empty D-FF nanotube *P_t_* is around ~0.14 C/m^2^, whereas for water-filled PNTs, after optimization, it is 0.133 C/m^2^ (see Table 3, columns 4 and 6); and for empty L-FF nanotubes, *P_t_* is around ~0.139 C/m^2^, whereas for water-filled PNTs, after optimization, it is 0.132 C/m^2^ only (see Table 4, columns 4 and 6). At the same time, the *P_t_* for separate water cluster after optimization is higher than that for filled D-FF and L-FF PNT and reaches ~0.15 C/m^2^ (Table 3, column 3), and ~0.148 C/m^2^ for L-FF (Table 4, column 3). This mismatch also demonstrates the strong effect of the electric field produced by the PNT. The maximum value of the corresponding electric field for both L-FF and D-FF (with polarization *P_t_* ~0.14 C/m^2^) can be estimated as *E = P_t_/* (εε_0_) ≈ 4.0 GV/m (for ε = 4 [18,24]—dielectric permittivity of the PNT, and ε_0_—dielectric vacuum constant). Such strong electric field oriented inside a cavity along the PNT’s *c*-axis induces the orientation of each water molecules dipole along this direction (Figure 5, Figure 6f,g, Figure 8 and Figure 9).

In this connection, it should be mentioned here that, in a carbon nanotube, water molecules form a solid-like structure under an applied electric field of 1 V/nm = 1 GV/m for simulation temperatures up to 350 K [64]. The authors Winarto W., Yamamoto E. Yasuoka K. of Ref. [64] suggested that the electric field induces a phase transition from liquid to ice-nanotube at temperatures as high as 350 K, and the electrostatic interaction within the ice-nanotube under an electric field is stronger than that in the absence of an electric field.

Another important point is that water cluster inside D-FF PNT acquiring per se strongly oriented polar properties and can reveal ferroelectric properties. Moreover, if we build longer models of optimized water ice clusters, we get extended needle-shaped strongly polarized ferroelectric-like elements that may have promising potential applications in various fields. As an example, Table 5 shows the results of calculations for more extended water clusters having even higher values of dipole moments and polarization. At the same time, here, we presented the calculation data using different methods, and we see that RM1 gives the highest values of the dipole moments (up ~100 Debye along PNT c-axis) and polarization (up ~0.174 C/m^2^). An important feature is that the value of the perpendicular components Dy and Py has different signs for D-FF and L-FF, corresponding to various orientations of the polar vector in accordance with chirality of PNT (see on Figure 8c and Figure 9c).

Some manifestations of ferroelectric properties in water structures are known, especially in their nanostructured form in various nanotube types, and under different influences [60,61,62,64,65]. In addition, it should be noted that the straightforward study of ferroelectric phenomena in water and ice tubular and one-dimensionally ordered nanostructures is actively carried out by some research teams. For example, Mikami F., Matsuda K., Kataura H. and Maniwa Y. in Ref. [65], novel ferroelectric properties of a new form of ice inside single-walled carbon nanotubes was investigated by molecular dynamics simulation. The authors called them “ice nanotubes” (ice NTs) and found them to consist of polygonal water rings stacked one-dimensionally along the nanotube axis. Mikami F., et al. in Ref. [65], ice NTs were revealed to show stepwise polarization with a significant hysteresis loop as a function of the external field strength. In particular, pentagonal and heptagonal ice NTs are found to be the world’s smallest ferroelectrics, with spontaneous polarization of around 1 μC/cm^2^ ~ 0.01 C/m^2^. In our case, for example, the *P_t_* value of water cluster in D-FF PNT is more than order of magnitude higher than this value (0.133 C/m^2^), and just greater up to ~0.15 C/m^2^ in the case of a such ice nanotube alone (and it is also higher for more long extended clusters; see Table 5). Thus, the presence of ferroelectric properties can be assumed as well. However, additional investigations are necessary.

All these findings show potential applications of nanotube encapsulating dielectric materials for the fabrication of the smallest ferroelectric devices. In turn, we assume that such and similar polar systems based on the tubular ice nanostructures, formed inside peptide nanotubes, have a great future for a variety of applications in many nanodevices.

Ferroelectric properties, in addition to polarization, also represent such a property as piezoelectricity, which has numerous practical applications [19,20,21,22,23,24,25,26,27,28,29,30,31,32]. Recently, it was shown by Bystrov V.S., Bdikin I.K., Singh B. in Ref. [18] that FF PNTs and also some AAs have rather high polarization values and, as a consequence, piezoelectric coefficients. Thus, the results obtained above not only mean that the presence of water/ice structures inside any AA and PNT increases and strengthens their polar properties, but also has very significant potential opportunities for the development of new types of nanostructures with enhanced piezoelectric properties.

## 4. Conclusions

The results obtained allow us to conclude that, on average, after optimizing the water/ice cluster enclosed in the inner hydrophilic cavity of the nanotubes, they acquire strongly anisotropic electrical properties, with significant magnitudes with dipole moments oriented mainly along the axis of the nanotubes. In this case, the average polarization values reach 0.16–0.17 C/m^2^ when calculated by any methods (the highest values are given by RM1, see Table 5) along the surface of the VdW surface.

In conclusion it must be stated, that the main results obtained include (1) after optimization, the embedded water molecular clusters have significantly larger dipole moments that are strongly oriented along D-FF and L-FF PNTs channel, while before optimization, it did not have such a defined orientation and had a very small dipole moments; (2) the structure organization of this water cluster has very significant and high changes—this water/ice cluster acquires helix properties, with the same helix step as D-FF PNT; (3) the dipole moment and polarization of the initial empty D-FF and L-FF PNT was increased and enhanced after optimization with the presence of the embedded water/ice cluster inside their cavity, particularly with the most optimal 21 H_2_O molecular cluster.

Such an obvious and drastic change modulated by the influence of D-FF properties and structural features was established here for the first time using direct modeling and calculations using DFT (VASP) and various semi-empirical quantum methods.

The whole set of the findings allowed us to assume the ferroelectric properties of such water clusters. However, the experimental confirmation of this assumption is still to be done. In this connection it must be noted that it is necessary to develop the investigation of the stability and the evolution of such clusters, especially, with the temperature, namely by doing MD simulations (similarly to that demonstrated by Andrade-Filho T. et al. in Ref. [36]), develop and perform the dielectric measurements of these structures (similarly to that reported by Bystrov V.S. et al. in Ref. [51]). In positive cases, these discoveries may lead in future to the development of unexpected applications and may also serve for further development in nanotechnological, medical and biological breakthroughs.

## Figures and Tables

**Table 1:** Lattice cell parameters for L-FF and D-FF PNT (from references [25,29,33] according to CCDC [39]).

	L-FF	D-FF
Space Group	P6_1_	P6_5_
*a*, Å	24.0709 (13)	23.9468 (14)
*b*, Å	24.0709 (13)	23.9468 (14)
*c*, Å	5.4560 (4)	5.4411 (2)
*V*, Å^3^	2737.7 (3)	2702.2 (2)

**Table 2:** Parameters of inner hydrophilic cavity of L-FF and D-FF PNTs.

Parameter	L-FF		D-FF	
	Initial	Opt (No Water)	Initial	Opt (No Water)
*a*, Å	24.0709	23.8308 (284)	23.9468	23.7877 (806)
*b*, Å	24.0709	23.8308 (284)	23.9468	23.7877 (806)
*c*, Å	5.456	5.4035 (861)	5.4411	5.4022 (7125)
R_0_, Å	12.236	12.091	12.102	12.075
R_1_, Å	15.271(698)	15.042 (076)	15.180 (569)	15.030 (688)
R_2_, Å	12.218(349)	12.098 (817)	12.135 (396)	12.075 (906)
Etot, eV	−1593.318267	−1657.643468	−1608.735638	−1657.600241

**Table 3:** Energy, dipole moment and polarization of D-FF PNTs and water clusters, computed using Austin Model 1 (AM1) restricted Hartree-Fock (RHF) method (HyperChem). Similar and close data were obtained by PM3 and RM1 methods.

Calculated Values		Extracted 42 H_2_O Cluster (21 H_2_O Per Unit Cell)		Two Coils of D-FF		
		Initial (*Ih)* Structure	After Optimization Inside D-FF	Initial D-FF Structure without H_2_O	With 21 H_2_O Per u.c. of Initial Structure	With 21 H_2_O Per u.c. after Optimization
1		2	3	4	5	6
Total energy, a.u.	*E_t_*	−534.5776	−537.78035	−1739.5256	−2271.06543	−2277.23035
	Δ*E_t_*	−3.20275 (−87.1513 eV)		–	−6.16492 (−167.7561 eV)	
Binding energy, eV	*E_b_*	−312.84856	−399.99734	−2265.27936	−2495.46785	−2663.21967
	Δ*E_b_*	−87.14878		–	−167.75182	
Dipole moment, D	*D_t_*	1.104	29.404	140.385	139.52	158.461
	*D_z_*	−0.876	−28.385	−140.349	−139.447	−158.441
Polarization, C/m^2^	*P_t_*	0.00569	0.15075	0.139927	0.119485	0.133218
	*P_z_*	−0.00451	−0.14554	−0.139892	−0.119423	−0.133201
VdW volume, Å^3^	*V*	647.8	650.55	3346.47	3894.86	3967.61

**Table 4:** Energy, dipole moment and polarization of L-FF PNTs and water clusters, computed using AM1 RHF method (HyperChem). Similar and close data were obtained by PM3 and RM1 methods.

Calculated Values		Extracted 42 H_2_O Cluster (21 H_2_O Per Unit Cell (u.c.))		Two Coils of L-FF		
		Initial (*Ih)* Structure	After Optimization Inside L-FF	Initial L-FF Structure without H_2_O	With 21 H_2_O Per u.c. of Initial Structure	With 21 H_2_O Per u.c. after Optimization
1		2	3	4	5	6
Total energy, a.u.	*E_t_*	−534.5776	−537.6803	−1739.0274	−2272.7630	−2278.8142
	Δ*E_t_*	−3.10268 (−84.42816 eV)		–	−6.05124 (−164.66275 eV)	
Binding energy, eV	*E_b_*	−312.8486	−397.2743	−2251.7229	−2541.6595	−2706.317
	Δ*E_b_*	−84.426		–	−164.657	
Dipole moment, D	*D_t_*	1.104	28.646	140.757	133.11	157.8331
	*D_z_*	−0.876	−28.386	−140.217	−130.279	−157.035
Polarization, C/m^2^	*P_t_*	0.00569	0.14824	0.1395	0.113128	0.13252
	*P_z_*	−0.00451	−0.14690	−0.13897	−0.110722	−0.13185
VdW volume, Å^3^	*V*	647.8	644.55	3365.6	3924.73	3972.63

**Table 5:** Dipole moments and polarization for water/ice cluster after optimization in D-FF and L-FF PNT, consisting from 126 H_2_O molecules (see Figure 8 and Figure 9), computed by various methods.

126 H_2_O Water/Ice Cluster after Optimization		Method Used (In RHF)					
		From D-FF			From L-FF		
		AM1	PM3	RM1	AM1	PM3	RM1
Dipole moment, Debye	Dt	95.92	97.355	100.575	94.982	97.226	99.533
	Dz	−93.496	−95.058	−98.130	−94.653	−96.787	−99.154
	Dy	−15.324	−14.816	−15.481	7.304	8.528	8.068
	Dx	14.978	14.912	15.695	2.991	3.521	3.188
Polarization, C/m^2^	Pt	0.166	0.1685	0.1741	0.1655	0.1698	0.1738
	Pz	−01618	−0.1645	−0.1699	−0.1653	−0.1690	−0.1731
	Py	−0.0265	−0.0256	−0.0265	0.0128	0.0149	0.0141
	Px	0.0259	0.0258	0.0259	0.0052	0.0061	0.0056
VdW Volume, Å^3^		1927.21			1910.28		
